# Integrating gene expression and protein-protein interaction network to prioritize cancer-associated genes

**DOI:** 10.1186/1471-2105-13-182

**Published:** 2012-07-28

**Authors:** Chao Wu, Jun Zhu, Xuegong Zhang

**Affiliations:** 1 MOE Key Laboratory of Bioinformatics and Bioinformatics Division, TNLIST and Department of Automation, Tsinghua University, Beijing 100084 PR, China; 2 Sage Bionetworks, Seattle, Washington, USA

## Abstract

**Background:**

To understand the roles they play in complex diseases, genes need to be investigated in the networks they are involved in. Integration of gene expression and network data is a promising approach to prioritize disease-associated genes. Some methods have been developed in this field, but the problem is still far from being solved.

**Results:**

In this paper, we developed a method, Networked Gene Prioritizer (NGP), to prioritize cancer-associated genes. Applications on several breast cancer and lung cancer datasets demonstrated that NGP performs better than the existing methods. It provides stable top ranking genes between independent datasets. The top-ranked genes by NGP are enriched in the cancer-associated pathways. The top-ranked genes by NGP-PLK1, MCM2, MCM3, MCM7, MCM10 and SKP2 might coordinate to promote cell cycle related processes in cancer but not normal cells.

**Conclusions:**

In this paper, we have developed a method named NGP, to prioritize cancer-associated genes. Our results demonstrated that NGP performs better than the existing methods.

## Background

To understand the roles they play in complex diseases, genes need to be investigated in the networks they are involved in [1]. Integration of gene expression and network data is a promising approach to prioritize disease-associated genes [2]. The prioritized genes can facilitate us to understand the molecular mechanism of disease and discover the promising candidates of drug targets.

Up to now, three main types of methods have been developed to prioritize disease-associated genes with gene expression and network data. The assumption of the first type is that the genes surrounded by differentially expressed (DE) genes in networks tend to be disease associated genes [3-7]. A recently published example of this type is the Heat Kernel Ranking method [7]. The second type is based on a network rewiring (NR) model to prioritize disease-associated genes [8-11]. In the NR model, the interactions of the candidate gene with other genes are assumed to be changed between normal and disease samples. The method by Taylor et al. [11] is a typical representative of this type. The third type considers the changes of gene interactions between normal and disease samples and their effects on gene expression to prioritize disease-associated genes [12,13]. The method of Regulatory Impact Factor or RIF [13] is a recently developed method of this type.

However, there are some drawbacks in the existing methods. For the first type of methods, networks are assumed to be static and may not reflect the specific condition under the study, and therefore it may produce many false positive results. For the second type of methods, it considers network variations but doesn’t consider their effects on gene expression. In some case, a top-ranked gene by this method may not play important roles in disease because its network variations may have little effects on the expression of its interacting genes. The third type of methods considers network variations and their effects on gene expression. However, in some situation, a disease-associated gene may lead to the differential expression of its interacting genes even there is no network rewiring. We call this situation as networked differential expression or ND for short.

In this paper, we have developed a method named Networked Gene Prioritizer (NGP) to prioritize cancer-associated genes. In our method, we assume that between compared samples, cancer-associated genes cause the differential expression of their interacting genes by NR and/or ND. We applied the proposed NGP method and three representative methods on 4 independent breast cancer patient microarray datasets and 3 independent non-small-cell lung cancer (NSCLC) patient microarray datasets. The compared methods include the Heat Kernel Ranking method [7], the method by Taylor et al. [11] and the RIF method [13]. We call them as HKR, the Taylor method and RIF, respectively, for the convenience of description. The results demonstrated that the proposed NGP method performs better than the compared methods. The top-ranked genes by NGP are stable between independent datasets and enriched in the cancer-associated pathways. Our results suggest that the top-ranked genes by NGP-PLK1, MCM2, MCM3, MCM7, MCM10 and SKP2 might coordinate to promote cell cycle related processes in cancer but not normal cells.

## Result

We applied NGP, HKR, the Taylor method and RIF on 4 independent breast cancer patient datasets and 3 independent NSCLC patient datasets (see Materials and Methods for the description of the methods and data). NGP can use two models: the NR model and the ND model (see Materials and Methods for details). We call them as NGP-NR and NGP-ND for short, respectively. RIF also has two models: RIF1 and RIF2 [13]. Two questions are asked when we compare their performances: 1). whether the top-ranked genes by the methods are stable between independent datasets; 2). whether the top-ranked genes are enriched in the cancer-associated pathways.

### Application on breast cancer patient datasets

We prioritized cancer-associated genes between ER positive and ER negative breast cancer patient samples.

First we investigated whether the methods can produce stable top 10, 25 and 50 genes between independent datasets. It is analyzed by the GSEA strategy and measured by *p* value (see Materials and Methods for detail). If the *p* values of the dataset pair are smaller than 0.005, the top ranking genes between the datasets are regarded as stable. As Additional file 1: Table S1, Additional file 2: Table S2 and Additional file 3: Table S3 show, HKR and NGP-ND produce stable top 10, 25 and 50 genes between all datasets while other methods fail. However, HKR’s results are not specific. It always ranked certain genes at top positions no matter what types of gene expression data (e.g., differential gene expression data of disease datasets, shuffled differential gene expression data of diseases datasets or differential gene expression data from the datasets of different diseases) were used (Additional file 4: Table S4). It has been demonstrated that a systematic bias that favors highly connected genes exists in many networked gene prioritization methods [3,14], and such systematic bias exists in HKR [3]. There is no obvious bias toward certain genes in the other methods because we can see that the top-ranking genes of these methods are unstable in either breast cancer or lung cancer datasets (Additional file 1: Table 1, Additional file 2: Table S2, Additional file 3: Table S3, Additional file 5: Table S5, Additional file 6: Table S6 and Additional file 7: Table S7).

Then we ranked genes according to their rank sum in independent datasets. The top 10 genes of the different methods are displayed in Table 1. The top 10, 25 and 50 genes of different methods were selected to conduct pathway analysis. The tool of pathway analysis is David functional annotation tools [15]. The pathway database is Reactome [16]. With Benjamin corrected *p* < 0.05 as threshold, we can see that some cell-cycle-related pathways are enriched in the top 10, 25 and 50 genes of NGP-ND (Table 2). The top ranking genes of the other methods are not enriched in any pathways. Reis-Filho and Pusztai had summarized the applications of microarray on the classification, prognostication, and prediction of breast cancer [17]. They found that when using the prognostic signatures that include many cell cycle and proliferation related genes, ER positive breast cancer can be classified into high proliferation group (about 50% samples) and low proliferation group (about 50% samples) but most of ER negative breast cancer patients are classified into high proliferation group (>95% samples). Their results suggest that the different functional status of cell cycle and proliferation processes may contribute to the difference between ER positive and ER negative breast cancer patients. The genes detected by NGP-ND are enriched in the cancer associated pathways between ER positive and ER negative breast cancer patients.

**Table 1:** The top 10 genes of different methods in breast cancer patient datasets

**Ranks**	**NGP-NR**	**NGP-ND**	**HKR**	**The Taylor method**	**RIF1**	**RIF2**
1	STAT3	PLK1	UBQLN4	HSPB1	FOXO1	CD247
2	EGFR	MCM2	SMAD9	RPA1	IGF1R	FGR
3	PDGFRB	MCM7	ESR1	S100B	RPS6KA3	LSM1
4	FOXO1	MCM3	CCDC85B	RNF11	ERBB2	IL2RB
5	AR	LCK	TP53	XPO1	TUBG1	CDKN2A
6	MCM10	CCNA2	GRB2	NFKB1	ENO2	RIPK2
7	CDKN2A	MCM10	ACTB	CPE	STAT5A	GZMB
8	SRPK1	SKP2	AR	SRPK1	CDC7	ITK
9	CCND1	LCP2	ACTA1	BCAP31	RBBP8	PRMT5
10	EPS8	CDC25C	CTNNB1	BCL2	HSP90AB1	COL1A1

**Table 2:** Pathways that the top ranking genes of different methods are enriched in in breast cancer patient datasets

**Top genes**	**Method**	**Pathway**	**Benjamin** ***p***
10	NGP-ND	REACT_152:Cell Cycle, Mitotic	3.00E-04
		REACT_383:DNA Replication	6.51E-04
		REACT_1538:Cell Cycle Checkpoints	0.020
25	NGP-ND	REACT_1538:Cell Cycle Checkpoints	3.84E-05
		REACT_152:Cell Cycle, Mitotic	1.13E-04
		REACT_383:DNA Replication	0.005
50	NGP-ND	REACT_152:Cell Cycle, Mitotic	3.62E-05
		REACT_1538:Cell Cycle Checkpoints	1.93E-04
		REACT_383:DNA Replication	0.0120

### Application on NSCLC patient datasets

We prioritized cancer-associated genes between lung cancer and normal samples.

When taking *p* < 0.005 as the threshold, NGP-NR, HKR and RIF2 produced stable top 10, 25 and 50 genes between independent datasets (Additional file 5: Table S5, Additional file 6: Table S6, Additional file 7: Table S7). However, as mentioned above, the top-ranking genes of HKR are not specific.

Then we ranked genes according to their rank sum in independent datasets. The top 10 genes of different methods are displayed in Table 3. The top 10, 25 and 50 genes were selected to conduct pathway analysis. It is showed that some cell-cycle related pathways are enriched in the top ranking genes of NGP-NR, the Taylor method, RIF1 and RIF2 (Additional file 8: Table S8). Sustaining proliferative signaling pushes the process of cell cycle into a different functional status in cancer cells compared to normal cells [18]. The pathway analysis result suggests that the genes top-ranked by NGP-NR, the Taylor method, RIF1 and RIF2 may be associated with the different functional status of cell cycle process between lung cancer and normal samples.

**Table 3:** The top 10 genes of different methods in NSCLC patient datasets

**Ranks**	**NGP-NR**	**NGP-ND**	**HKR**	**The Taylor method**	**RIF1**	**RIF2**
1	PLK1	PCNA	UBQLN4	CDC7	PLK1	SPARC
2	MCM7	TGFBR2	SMAD9	MCM7	CDC6	CDC20
3	CDC7	SYK	TP53	SKP2	MCM7	MCM2
4	BRCA1	MCM2	TGFBR1	MCM3	MCM3	CDC6
5	EGFR	GPRASP1	ACTA1	PLK1	CDC7	MCM3
6	MCM2	CAV1	GRB2	TUBB	MCM10	CD247
7	NDC80	CCNA2	CTNNB1	MCM10	MCM2	CD4
8	MAPK1	JAK2	EP300	GAB2	YES1	CDC7
9	MCM3	STAT5B	ACTB	MCM2	S100B	MCM7
10	SKP2	MAPK1	CCDC85B	CD247	DHX9	NDC80

**Figure 1:**
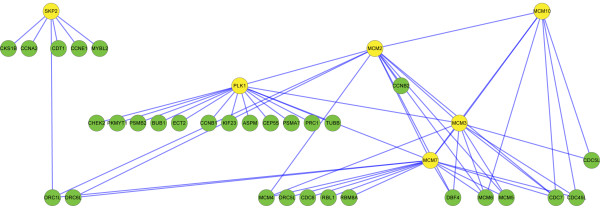
PLK1-MCM complex-SKP2 subnet in breast cancer patient datasets.

**Figure 2:**
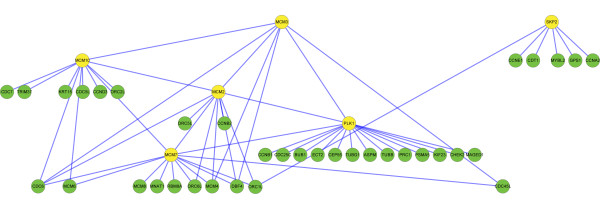
PLK1-MCM complex-SKP2 subnet in NSCLC patient datasets.

**Figure 3:**
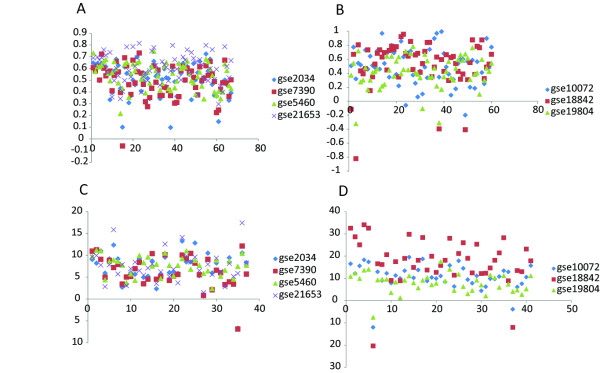
**Expression correlation and differential expression of genes in the PLK1-MCM complex-SKP2 subnets.A,** In NGP-ND, PPIs are weighted by the absolute average of Spearman coefficient of interacting genes’ expression in ER positive and ER negative samples. The weights of all the PPIs in the PLK1-MCM complex-SKP2 subnet of breast cancer patient datasets are displayed. **B,** In NGP-NR, PPIs are weighted by the absolute difference of Spearman coefficient of interacting genes’ expression in lung cancer and normal samples. The weights of all the PPIs in the PLK1-MCM complex-SKP2 subnet of NSCLC patient datasets are displayed. **C,** The differential expression of genes in the PLK1-MCM complex-SKP2 subnet of breast cancer patient datasets is displayed by *–log(p)*, where *p* is estimated by *t* test. Genes that are up regulated and down regulated in ER negative samples are displayed in upper and lower right quadrant, respectively. **D,** The differential expression of genes in the PLK1-MCM complex-SKP2 subnet of NSCLC patient datasets is displayed by *–log (p)*. Genes that are up regulated and down regulated in lung cancer samples are displayed in upper and lower right quadrant, respectively.

It is interesting to see that the top ranking genes of NGP-ND in breast cancer patient datasets and the top ranking genes of NGP-NR in NSCLC patient datasets are overlapped (Table 1 and 3). We selected the overlapped genes-PLK1, MCM2, MCM3, MCM7, MCM10 (ranked 13 by NGP-NR in NSCLC patient datasets) and SKP2 to investigate their functions in cancer. In either breast cancer or NSCLC patient datasets, we screened their DE subnets (see methodology of NGP in Materials and Methods for the definition of “DE subnet”) in independent datasets and selected PPIs that appeared in more than one datasets to construct a network (Figures 1 and 2). We call the network as PLK1-MCM complex-SKP2 subnet for short. Thirty one genes are overlapped in the PLK1-MCM complex-SKP2 subnets of breast cancer patient datasets (37 genes in total) and the one of NSCLC patient datasets (42 genes in total). David functional annotation analysis showed 75% genes of the PLK1-MCM complex-SKP2 subnet in breast cancer patient datasets and 70.7% genes of the PLK1-MCM complex-SKP2 subnet in NSCLC patient datasets are involved in the Reactome pathway of “Cell Cycle, Mitotic”. Expression of the interacting genes in the PLK1-MCM complex-SKP2 subnet of breast cancer patient datasets is positively correlated in ER positive samples and ER negative samples (Figure 3A). The interacting genes’ expression in the PLK1-MCM complex-SKP2 subnet of NSCLC patient datasets is weakly positively correlated or negatively correlated in normal samples but positively correlated in lung cancer samples (Figure 3B). Expression of the genes in the PLK1-MCM complex-SKP2 subnet of breast cancer patient datasets is up regulated in ER negative samples (Figure 3C); expression of the genes in the PLK1-MCM complex-SKP2 subnet of NSCLC patient dataset is up regulated in lung cancer samples (Figure 3D). These results suggest that the genes in the PLK1-MCM complex-SKP2 subnets coordinately work in breast cancer and lung cancer samples but not normal samples.

MCM2-7 are eukaryotic replicative helicases, they unwind DNA double strands in DNA replication process [19]. Ge XQ et al. [20] demonstrated that: 1). Inhibiting the expression of MCM5 with siRNA will reduce chromatin bound MCM2, MCM3, MCM5, MCM6, MCM7 ~50%, but this will not obstruct DNA replication in normal cells; 2). When cells suffer from replicative stress and replicative forks are slowed or stalled, this will make cells not survive. Their result suggests that cells may depend on excess MCM2-7 to efficiently replicate DNA when replicative forks are slowed or stalled. PLK1 is a marker of cellular proliferation, and plays important roles in cancer development [21]. High level of PLK1 expression is detected in NSCLC and other tumors [22]. Trenz K et al. [23] showed that: 1). Plx1, the Xenopus orthologue of Plk1, is dispensable in unchallenged chromosomal DNA replication; 2). When cells suffer from DNA replication stress and forks are stalled, Plx1 will bind with MCM2-7 to promote DNA replication. The genome of tumor cells is highly unstable. Many DNA lesions exist in tumor genome and they would normally interfere with replication progression. The coordination of PLK1, MCM complex and their interacting genes (Figure 1 and 2) might driver DNA replication forward, overcoming the effects of replication stress in breast cancer and lung cancer cells.

SKP2 plays roles in the transition of cell cycle and behaves as an oncogene [24]. Lin HK et al. demonstrated that under the oncogenic condition (e.g., aberrant proto-oncogenic signals or inactivation of tumor suppressor genes) inactivation of Skp2 will cause cell senescence, but in normal condition inactivation of Skp2 will not influence the senescence of cell [25]. With the work of Lin HK et al. and our results, it is suggested that SKP2 promotes the transition of cell cycle in cancer but not normal cells.

With above results, it is suspected that PLK1, MCM complex, SKP2 and some of their interacting genes (Figure 1 and 2) may play important roles to promote cell cycle related processes in cancer but not normal cells. They could be considered as the promising candidates of drug targets for cancer therapy.

## Discussion

In this paper, we have developed a method named Networked Gene Prioritizer (NGP), to prioritize cancer-associated genes. We compared the performances of NGP with 3 existing methods—HKR, the Taylor method and RIF. The results showed NGP performs better than the compared methods. The different models (NR and ND) make it be able to produce stable top-ranking genes and detect genes in the cancer associated pathways in breast cancer and lung cancer patient datasets. RIF2 succeeds in producing stable top-ranking genes and detecting genes in the cancer associated pathways in breast cancer patient datasets. The other methods fail in breast cancer and lung cancer patient datasets.

NGP outperforms HKR, the Taylor method and RIF because: 1). In HKR, network is static and may not reflect the specific condition under the study. So the systematic bias favoring highly connected genes makes HKR always rank certain genes at top positions no matter what gene expression data is input. In NGP, the PPIs are weighted by gene expression correlations to reflect the network dynamics under the study. 2). The Taylor method considers network variations but doesn’t consider their effects on gene expression. The top prioritized gene by this method may not play important roles in the disease because its network variations may have little effects on the expression of its interacting genes. In NGP-NR, the effects of network variations are measured by the differential expression of interacting genes. 3). Although RIF and NGP-NR both consider network variations and their effects on gene expression to prioritize disease-associated genes, they adopt different models to integrate gene expression and network. In RIF, prioritization of the candidate gene depends on the network variations of the candidate gene with DE interacting genes and their effects on the expression of the DE interacting genes. But the GSEA strategy facilitates NGP-NR to consider the network variations of the candidate gene with all its interacting genes and their effects on the expression of the interacting genes. NGP-NR considers more information about candidate genes’ interacting genes than RIF. Moreover, besides the situation RIF considered (a cancer-associated genes cause the differential expression of their interacting genes by NR); NGP considers one more situation that a disease-associated gene’s dysregulation of expression can lead to the differential expression of its interacting genes when there is no network rewiring. NGP-ND is developed to prioritize genes in this situation.

However, in our experiments, we can see that the two models of NGP (NR model and ND model) do not work equally well on different applications. By their definition, we can see that the two models aim to detect the different types of cancer-associated genes. The problem of NGP is that how to choose a proper model (NGP-NR, NGP-ND, or them both) to prioritize cancer-associated genes when we don’t know the molecular interaction mechanisms of cancer-associated genes with their interacting partners between compared conditions. Our experiences suggest that a judgment can help us find the proper model: whether the model adopted can produce stable top ranking genes between independent datasets.

Additional effort is needed to improve NGP. Genetic studies identify genetic variation locations (e.g., copy number variations or SNPs) involved in disease and provide candidate genes associated with the disease. By investigating the impacts of candidate genes’ variations on their own and their target genes’ expression, people can discover disease-associated genes. Recently, Akavia UD et al. integrated gene expression and copy number variation data to uncover drivers of cancers [26]. With some modifications NGP can also be used to rank candidate genes inferred from other studies such as genetic studies.

## Conclusions

In this paper, we have developed a method named NGP, to prioritize cancer-associated genes. The results showed NGP performs better than the existing methods.

## Methods

### Data and data pre-processing

Seven microarray datasets are used in this paper (Additional file 9: Table S9), including 4 independent breast cancer patient datasets: gse5460 [27], gse7390 [28], gse21653 [29] and gse2034 [30], and 3 independent NSCLC patient datasets: gse19804 [31], gse18842 [32], gse10072 [33]. All datasets were downloaded from Gene Expression Omnibus (GEO) database [34]. Each microarray dataset was processed as following: at first, raw data were processed by RMA [35]; then, the samples were classified into different classes (e.g., ER positive and ER negative in breast cancer patient datasets). However, in the breast cancer patient datasets with histological type information, only the samples of ductal type were selected for further analyses.

For each dataset, gene expression profiles were processed as following: at first, the ambiguous probe sets (mapped to more than one gene) were filtered out; then, differential expression analysis of probe sets between compared samples was conducted (*t* test, two tails, unequal variation); at last, for each gene, the probe set with most significant *p* value was selected and the probe set’s expression level was assigned as the gene’s expression level. The reason to assign gene expression in this way is that we assume gene expression has significantly changed between compared samples. Thus, the expression of the probe set with most significant *p* value between compared samples is the best candidate to represent the expression of the gene.

Protein-protein interactions were downloaded from HPRD [36] database (Release 9). Excluded self-interactions, 9453 proteins and 36867 PPIs were selected.

### Pre-selection of candidate genes

Candidate genes were pre-selected as hub genes in affy 133a PPI network; the hub genes are genes with more than 15 interacting genes in the PPI network; affy 133a network is the PPI network constructed by the genes in both Affymetrix HGU-133A chip and PPIs of HPRD. We pre-selected candidate genes in this way because: 1). It is believed that genes with many interacting partners in the network tend to play important roles in cell, for example, they tend to be essential genes or disease-associated genes [37]. 2). In NGP, it is required that a candidate gene should have more than 15 interacting genes in the network (please see NGP methodology for detail). To make sure the different methods start from the same candidate genes set, the hub genes are determined as the genes with more than 15 interacting genes in PPI network. 3). PPI network is constructed by genes both in microarray and PPIs of HPRD. Two types of microarray datasets, Affymetrix HGU-133A and Affymetrix HGU-133Plus2, are used in this paper. Affymetrix HGU-133Plus2 covers the probe sets of Affymetrix HGU-133A. In the experiments on different types of microarray datasets, different PPI networks will be used. To make sure the experiments of different types of microarray datasets start from the same candidate gene sets, the hub genes are determined as the hub genes in affy 133a PPI network. Totally, 953 genes were selected. Then in each method, the pre-selected genes were further processed to get priority list of the method.

### Brief introduction of HKR, the Taylor method and RIF

HKR prioritizes genes based on the differential expression of their neighboring genes [3]. A characteristic of HKR is that it takes the random walk strategy to detect candidate genes’ neighboring genes in the network. Two inputs-network and gene differential expression are required to run HKR. The output of HKR is a gene rank list. In our experiments, network is the HPRD network. Gene differential expression is assigned as *–log(p)*,where *p* was estimated by *t* test of gene expression between compared samples (two-tailed, unequal variation). Please refer PINTA [7] for more information.

The Taylor method prioritizes genes based on the network variations of candidate genes with their interacting genes between compared samples [11]. Gene expression correlation is used to measure the dynamic action of the PPIs. The difference of gene expression correlations of the PPIs in compared samples is used to test whether the interactions have been varied. In our experiments, the HPRD network was used. Pearson Coefficient was used to measure the dynamic action of the PPIs. Candidate genes were ranked by averaged absolute difference of Pearson Coefficient of the candidate genes with their interacting genes between compared samples. Please refer the Taylor method [11] for more information.

In RIF, prioritization of a candidate gene depends on the network variations of the candidate gene with DE interacting genes and their effects on the expression of these DE interacting genes. Two alternative measures of RIF are computed as equation 1 and 2. Then, the RIF1 and RIF2 of all the candidate genes are Z-score normalized, respectively.

(1) $$RIF1_{i}=\frac{1}{n_{\text{DE}}}\sum_{j=1}^{j=n_{\text{DE}}}\frac{1}{2}\left(e1_{j}^{2}-e2_{j}^{2}\right){\left(r1_{\text{ij}}-r2_{\text{ij}}\right)}^{2}$$

(2) $$RIF2_{i}=\frac{1}{n_{\text{DE}}}\sum_{j=1}^{j=n_{\text{DE}}}\left[{\left(e1_{j}\times r1_{\text{ij}}\right)}^{2}-{\left(e2_{j}\times r2_{\text{ij}}\right)}^{2}\right]$$

where *n*_*DE*_ is the number of DE genes that candidate gene *i* interacted; *e*1_*j*_ and *e*2_*j*_ are the average expression of gene *j* in compared samples, respectively; *r*1_*ij*_ and *r*2_*ij*_ are Pearson Coefficient between gene *i* and *j* in compared samples, respectively.

In our experiments, the HPRD network was used. Gene differential expression was measured by *t* test (two-tailed, unequal variation). False discovery rate (FDR) was used to correct for multiple comparisons. DE genes were selected by FDR < 0.01. We ranked candidate genes by their normalized RIF1 scores and RIF2 scores.

### Networked Gene Prioritizer

**Figure 4:**
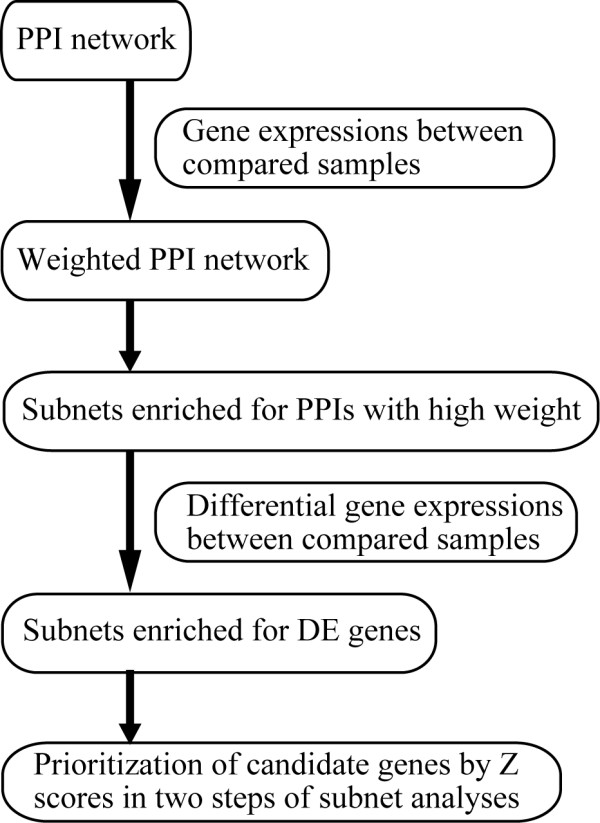
**Flowchart of NGP.** PPI: protein-protein interaction; DE genes: differentially expressed genes.

We introduced NGP with a flowchart (Figure 4).

### PPI network

PPI network was constructed by genes both in microarray and in PPIs of HPRD. It is defined as *G = (V,E)*, where *V* is the set of genes, *E* is the set of interactions.

### Weighted PPI network

PPIs were weighted differently in NR and ND models. In NR model, it is assumed that cancer-associated genes cause the differential expression of its interacting genes by changing the genes it interacts. In ND model, it is assumed that cancer-associated gene’s dysregulation of expression can lead to the differential expression of its interacting genes when there is no network rewiring. We used gene expression correlation to measure the dynamic action of the PPIs in different samples. The difference of gene expression correlations between compared samples was used to test whether the PPI has been changed. So in NGP-NR, PPIs were weighted by absolute difference of Spearman coefficient of the interacting genes in compared samples ($\left|\Delta r_{E_{\text{ij}}}\right|$). In NGP-ND, the consistently high gene expression correlations in compared samples suggest there is no network rewiring between the compared samples. So the PPIs were weighted by absolute average of Spearman coefficient of the interacting genes in compared samples ($\left|\bar{r_{E_{\text{ij}}}}\right|$). $\left|\bar{r_{E_{\text{ij}}}}\right|$ and $\left|\Delta r_{E_{\text{ij}}}\right|$ were calculated by equations 3–5.

(3) $$r_{E_{\text{ij}}}=\frac{\sum_{k}\left(x_{\mathrm{ik}}-\bar{x_{i}}\right)\left(x_{\mathrm{jk}}-\bar{x_{j}}\right)}{\sqrt{\sum_{k}\left(x_{\mathrm{ik}}-\bar{x_{i}}\right)\sum_{k}\left(x_{\mathrm{jk}}-\bar{x_{j}}\right)}}$$

where *E*_*ij*_ is the PPI between gene *V*_*i*_ and gene *V*_*j*_; *k* is the *kth* sample; *V*_*i*_ and *V*_*j*_ are ranked by their expressions in the samples respectively, and *X*_*jk*_ is the rank of *V*_*i*_ of *kth* sample, *X*_*ik*_ is the rank of *V*_*j*_ of *kth* sample; $\bar{x_{j}}$, $\bar{x_{j}}$ are the average ranks of *V*_*i*_ and *V*_*j*_ in the samples, respectively.

(4) $$\left|\bar{r_{E_{\text{ij}}}}\right|=\frac{1}{2}\left|r_{E_{ij1}}+r_{E_{ij2}}\right|$$

(5) $$\left|\Delta r_{E_{\text{ij}}}\right|=\left|r_{E_{ij1}}-r_{E_{ij2}}\right|$$

Where $r_{E_{ij1}}$ and $r_{E_{ij2}}$ represent the Spearman coefficients of *E*_*ij*_ in compared samples respectively.

However, we first filtered out the PPIs that had been changed between compared samples in NGP-ND. We calculated the $\left|\Delta r_{E_{\text{ij}}}\right|$ of each PPI, then we permuted samples labels (e.g., lung cancer or normal) 10000 times and generated a random $\left|\Delta r_{E_{\text{ij}}}\right|$, at last the PPIs whose $\left|\Delta r_{E_{\text{ij}}}\right|$ are larger than 90% random $\left|\Delta r_{E_{\text{ij}}}\right|$ were filtered out.

### Subnets enriched for the PPIs with high weight

In this step, the genes with more than 15 interacting genes in the weighted network were selected as candidate genes. Candidate genes’ subnets consist of them and their interacting genes. Then the subnets were screened for detecting the subnets enriched for the PPIs with high weight. It was conducted by the GSEA [38] strategy: at first, PPIs in weighted network and candidate gene’s subnet (*S*) were regarded as background set and objective set, respectively; next, PPIs in background set were ranked by their weight; then, enrichment score (*ES*) of the subnet was calculated as the maximum deviation of *P*_*hit*_(*S*, *i*) and *P*_*miss*_(*S*, *i*) when *i* walked in the ranked PPIs as following:

(6) $$P_{\text{hit}}\left(S,i\right)=\sum_{E_{j}\in S,j\leq i}\frac{{\left|r_{j}\right|}^{P}}{N_{R}},$$

where

(7) $$N_{R}={\underset{E_{j}\in S}{\sum }\left|r_{j}\right|}^{P}$$

(8) $$P_{\text{miss}}\left(S,i\right)=\sum_{E_{j}\in S,j\leq i}\frac{1}{N-N_{H}}$$

where *E*_*j*_ is the *jth* PPI in the ranked PPIs; *r*_*j*_ is the weight of the *jth* PPI in background set; *P* is a parameter and set as 1; *N* is the number of PPIs in *E*; *N*_*H*_ is the number of PPIs in the subnet *S*.

The candidate genes were selected as the genes with more than 15 interacting genes in the weighted network because in GSEA the min size of objective sets is 15. Statistical significance of *ES* was estimated with Z score. It was conducted in this way: at first, we permuted the weights of PPIs in weighted network and calculated *ES*; next, we conducted the permutation 1000 times to generate a random *ES* set ; then we estimated the Z score of *ES* of the subnet (*Z*_*S*_) as

(9) $$Z_{s}=\frac{ES-\bar{ES}}{S^{'}}$$

Where $\bar{ES}$ is the mean of the random *ES* set; *S*′ is the standard deviation of the random *ES* set.

At last, we trimmed the subnet by filtering out its PPIs that didn’t contribute to *ES.*

### Subnets enriched for differentially expressed genes.

The trimmed subnets (*S*_*trimmed*_) were further screened for detecting the subnets enriched for DE genes. It was also conducted by the GSEA strategy: at first, genes in microarray (*L*) and the trimmed subnet were regarded as background set and objective set respectively; next, genes in the background set were ranked according to *–log(p)*,where *p* was estimated by *t* test of gene expression between compared samples (two-tailed, unequal variation); then, *ES* of *S*_*trimmed*_ was calculated as the maximum deviation of *P*_*hit*_(*S*_*trimmed*_, *i*) and *P*_*miss*_(*S*_*trimmed*_, *i*) when *i* walked in the ranked genes.

(10) $$P_{\text{hit}}\left(S_{\text{trimmed}},i\right)=\sum_{g_{j}\in S_{\text{trimmed}},j\leq i}\frac{{\left|r_{j}\right|}^{P}}{M_{R}},$$

where

(11) $$M_{R}={\sum_{g_{j}\in S_{\text{trimmed}}}\left|r_{j}\right|}^{P}$$

(12) $$P_{\text{miss}}\left(S_{\text{trimmed}},i\right)=\sum_{g_{j}\in S_{\text{trimmed}},j\leq i}\frac{1}{M-M_{H}}$$

where *g*_*j*_ is the *jth* gene in the ranked genes; *r*_*j*_ is the magnitude of differential expression of the *jth* gene; *P* is a parameter and set as 1; *M* is the number of genes in *L*; *M*_*H*_ is the number of genes in *S*_*trimmed*_.

Statistical significance of *ES* of the trimmed subnet was estimated by Z score. The Z score was estimated on the similar way that we did in “Subnets enriched for PPI with high weight” section.

At last, the trimmed subnet (*S*_*trimmed*_) was further trimmed by filtering out its genes that didn’t contribute to *ES*. The trimmed *S*_*trimmed*_ is defined as DE subnet.

### Prioritization of candidate genes

Candidate genes were prioritized according to the sum of Z scores they got in above two steps of subnet analyses (equation 11). To make them comparable, Z scores of different types of subnets were Z-score normalized respectively.

(13) $$ZS_{combined\_i}=Z_{s\_i}^{'}+Z_{\text{trimmed}\_i}^{'}$$

*i* represents the *ith* candidate gene, *Z*′_*s*_*i*_ is the normalized *Z*_*S*_ of the *ith* candidate gene, *Z*_*trimmed*_*i*_^’^ is the normalized *Z*_*trimmed*_ of the *ith* candidate gene.

### Stability analysis of top-ranking genes between independent datasets

The aim of stability analysis of top-ranking genes between independent datasets is to investigate whether the methods can produce stable top-ranking genes between independent datasets. For the priority lists in two datasets A and B, if most of the top-ranking genes in A are also the upper ranking genes in B, we regard the top-ranking genes in A are stable in B. If the top-ranking genes in A are stable in B and vice versa, we regard the top-ranking genes are stable between A and B. The stability analysis was also conducted by the GSEA strategy. Objective set was the top-ranking genes in one dataset (e.g., A). Background set was the candidate genes in the other dataset (e.g., B). The parameter *P* of GSEA was set as 0 and the random time was set as 1000. The significance of the observed *ES* was measured by nominal *p*, which was estimated by comparing the observed *ES* with a set of randomized *ES*. Please refer the original paper of the GSEA method [38] for the detail of the estimation of nominal *p*.

## Competing interests

The authors declare that they have no competing interests.

## Authors' contributions

CW collected the data, designed and conducted the study, and drafted the manuscript. JZ and XZ participated in the design of the study and helped to draft the manuscript. All authors read and approved the final manuscript.

## Supplementary Material

Additional file 1 **Table S1.** Stability of the top 10 genes in different methods between breast cancer patient datasets. Click here for file

Additional file 2 **Table S2.** Stability of the top 25 genes in different methods between breast cancer patient datasets. Click here for file

Additional file 3 **Table S3.** Stability of the top 50 genes in different methods between breast cancer patient datasets. Click here for file

Additional file 4 **Table S4.** The top 10 genes of HKR on disease datasets and shuffled disease datasets. Click here for file

Additional file 5 **Table S5.** Stability of the top 10 genes in different methods between NSCLC patient datasets. Click here for file

Additional file 6 **Table S6.** Stability of the top 25 genes in different methods between NSCLC patient datasets. Click here for file

Additional file 7 **Table S7.** Stability of the top 50 genes in different methods between NSCLC patient datasets. Click here for file

Additional file 8 **Table S8.** Pathways that the top ranking genes of different methods are enriched in in NSCLC patient datasets. Click here for file

Additional file 9 **Table S9.** Information of the samples used in this paper. Click here for file
